# Medical decision making beyond evidence: Correlates of belief in complementary and alternative medicine (CAM) and homeopathy

**DOI:** 10.1371/journal.pone.0284383

**Published:** 2023-04-21

**Authors:** Leonie Aßmann, Tilmann Betsch

**Affiliations:** Department of Psychology, Faculty of Education, University of Erfurt, Erfurt, Germany; National Sun Yat-sen University, TAIWAN

## Abstract

Many people believe in and use complementary and alternative medicine (CAM) to address health issues or prevent diseases. Empirical evidence for those treatments is either lacking or controversial due to methodological weaknesses. Thus, practitioners and patients primarily rely on subjective references rather than credible empirical evidence from systematic research. This study investigated whether cognitive and personality factors explain differences in belief in CAM and homeopathy. We investigated the robustness of 21 predictors when examined together to obtain insights into key determinants of such beliefs in a sample of 599 participants (60% female, 18-81 years). A combination of predictors explained 20% of the variance in CAM belief (predictors: ontological confusions, spiritual epistemology, agreeableness, death anxiety, gender) and approximately 21% of the variance in belief in homeopathy (predictors: ontological confusions, illusory pattern perception, need for cognitive closure, need for cognition, honesty-humility, death anxiety, gender, age). Individuals believing in CAM and homeopathy have cognitive biases and certain individual differences which make them perceive the world differently. Findings are discussed in the context of previous literature and in relation to other unfounded beliefs.

## Introduction

Imagine you have a sore throat, runny nose, and itchy eyes—you might have caught a cold. You think it is not yet necessary to see a doctor, but you want to take something for relief and that helps your body to get better. What is your choice of remedy? There are treatments provided by conventional medicine as well as by complementary and alternative medicine (CAM). CAM differs substantially from conventional medicine [1], although a clear definition of CAM is lacking [2] and quite difficult to achieve since CAM comprises approximately 400 procedures that differ widely in methodical approaches [3]. In general, CAM treatments include not only remedies but also a wide range of practices and other modalities—the offers are highly diverse. Some forms can be received as delivered by a practitioner (e.g., acupuncture or chiropractic treatments), whereas others involve self-care practices, i.e., homeopathic remedies, herbal medicines, and vitamins [4]. All treatments are commonly not provided within conventional medicine settings, since they do not adhere to the dominant biomedical model of health and evidence-based health care [5–7]. Nevertheless, many people use CAM to address health issues and prevent diseases. This also applies for people who do not refuse conventional medicine [8, 9]. Hence, the remedies are widely used as complementary treatments to conventional medicine but sometimes also as alternative treatments.

The belief in CAM and use of CAM treatments are prominent all over the globe. In India, CAM treatments are part of public health care [10]. In Europe, e.g., Germany and Switzerland, they are covered by public health insurance [11, 12] and universities provide courses in Traditional Chinese Medicine, Anthroposophical Medicine, and homeopathy [13–15]. In other western countries, e.g., in the US, UK, and Australia, a small but considerable number of people are attracted to CAM treatments [12]. In Europe, approximately 26% of the general population have experience using CAM. The use depends highly on the country [16]. Especially in Germany, various forms of CAM are used frequently. The most favored form is homeopathy: 55% have experience with the use of homeopathic remedies. Only approximately one in four (26%) report a refusal in using homeopathic treatment [17]. The use of homeopathy often includes not only visits to homeopaths but also purchasing over-the-counter homeopathic medicines [12]. For example, German pharmacies turned over approximately 542 million Euros with homeopathic medicines in 2018 [18]. In many cases, the use of homeopathy goes along with use of other CAM methods [19, 20] and sometimes also with rejection of conventional medicine (e.g., vaccinations [6]).

### Evidence-based medicine, science, CAM, and homeopathy

One reason for the popularity of CAM and homeopathy is that such treatments are perceived to be low risk with regard to negative side effects. On the other hand, it is questionable whether they have any effects at all: quite often they have not been empirically tested [6] nor is there empirical evidence for their usefulness beyond placebo effects (e.g., homeopathy [7]). Hence, the main ‘evidence’ for CAM treatments is merely anecdotical [2, 21]. Practitioners and patients primarily rely on subjective references—successful treatment experiences reported by family, friends, and colleagues—rather than credible empirical evidence from systematic research.

However, empirical evidence should be used as a guideline in health-related decision making along with other considerations such as an evaluation of risks [22]. Evidence-based medicine is an approach that combines “individual clinical expertise with the best available external evidence from systematic research” [23]. This has become the most prevalent norm not only for medical decision making but also for clinical practice guidelines [3, 24]. In conventional medicine, the demonstration that a treatment is relatively safe and effective is a necessary requirement before it is included in public health care [21]. This demonstration is generally accomplished through empirical studies with randomized controlled trials (RCTs). Without such research, both patients and practitioners alike tend to attribute improvements in health status as valid treatment effects. Consequently, CAM “embraces subjective, emotive truth criteria, whereas its detractors demand objective evidence” [21]. In addition, the hypothesized mechanisms are in conflict with those accepted by science. This is fundamental in studies concerning homeopathic remedies. The theoretical explanations for the effectiveness of homeopathy (i.e., the similarity rule and exponentiation) violate fundamental principles of natural sciences. Therefore, it might be considered questionable per se to test “whether magic works” [25]. Notwithstanding its unsound theoretical background, studies on the effects of homeopathic treatments have been accumulating during the recent decades. The majority are methodologically severely flawed. Studies that are high quality and adhere to scientific standards find no effects of homeopathy beyond placebo effects (e.g. [26–28]). Further, numerous reviews and meta-analyses converge in showing that there is no empirical evidence that homeopathic remedies have any effect beyond control group level [28–33]. Rather, placebo effects are seen as the most obvious cause for the effects experienced after homeopathic treatments [3, 32–34].

### Determinants and correlates of beliefs in CAM and homeopathy

When decisions for CAM and homeopathy treatments are beyond evidence, the key question is: why do so many people still believe in CAM as well as homeopathy despite the presence of contrary data? This question is important not only for academia but also for applied contexts and especially public health, because incorrect health-related decisions do not only imply economic loss but can also have serious consequences for the individual health status. Those consequences arise through delayed conventional treatments or unexpected side effects of CAM treatments. Thus, it is necessary to understand what fosters such beliefs.

In many studies, the focus was on socio-demographic variables. It was found that primarily middle-aged [8], well-educated women use CAM [16, 35] and homeopathic treatments, in particular [4, 8, 19, 35, 36]. In addition, reasoning skills have also been examined in relation to CAM belief and use. In principle, human information processing is often biased by social and emotional factors and motivated to confirm existing beliefs [6, 37, 38]. This is, because rational and experiential knowledge systems work in parallel. Both depend on each other [22] and existing information (scientific and non-scientific) is needed to process new (scientific) information, and to decide if it is accepted, rejected or assimilated [39]. Also, decision making is affected by reasoning skills and cognitive style [40–42]. Beyond situational factors evoking different thinking styles, there are also stable individual differences in the tendency to overcome intuitive responses through additional reflection [42]. Those differences in the willingness to reason analytically oppose the susceptibility to biases in decision making [6]. Decisions for health-related treatments involve uncertainty and appropriate weighting of probabilities. Dealing with numerical information is often easier for people with higher numeracy and a tendency to reason analytically. In the context of health, people with an analytic cognitive style evaluate treatments more favorably when they are in line with available evidence. Thus, analytical thinking style was negatively related to all forms of CAM, having a significant relationship with homeopathy [6]. In contrast, an intuitive cognitive style is associated with impulsive decision making—thus, it is connected to more incorrect evaluations of CAM treatments [4, 7]. In addition, evidence-based health decisions require a certain understanding of science and scientific methods. Thus, an accurate understanding of the evidence around CAM and homeopathy is related to individual differences in scientific reasoning. This was a predictor not only for CAM belief but also for the use of CAM [2, 9]. It also contributes to an understanding of causality. One of the most common and invalid assumptions in (medical) decision making is confusing correlation or coincidence with causation [21]—for instance, attributing a random effect to a treatment without testing for a causal relationship. Additionally, people tend to perceive illusory patterns in random events [43]. The automatic tendency to see meaningful connections between stimuli arises from the fact that people strive to understand the world. However, these otherwise functional processes can be disrupted, so that connections are mistakenly seen between stimuli that are actually unrelated. Thus, in Illusory Pattern Perception, coherent and meaningful connections are seen in a set of random stimuli. These include the perception of false correlations [43, 44].

Not only cognitive factors influence our perceptions and decision making. Personality can shape our beliefs as well. In the context of CAM, openness to new experience is an associated personality dimension [4, 7, 45]. Open individuals often favor “a personal, emotional, and spiritual approach to health decisions” and thus reject objective scientific knowledge [6]. Research findings for the relationship between CAM and neuroticism were mixed—some studies found a positive, others a negative relationship [7].

Certain beliefs—more specifically, unfounded beliefs such as paranormal beliefs or conspiracies—are directly related to CAM belief. Previous studies showed that CAM believers tend to also believe in paranormal phenomena and conspiracies [8, 46, 47]. Paranormal beliefs are usually built on a magical worldview without reasoned review [48], which is shared by CAM. Both belief forms advocate emotional criteria for truth instead of empirical data and logical considerations. Another belief, namely spirituality, is closely related to paranormal beliefs and religiosity, and also associated with being a CAM user [4, 45, 49, 50]. Lindeman [46] found that CAM belief could be best explained by intuitive reasoning, paranormal beliefs, and ontological confusions. Ontological confusions are defined as category mistakes in which properties of living and lifeless entities are mixed [51].

### This study

Taken together, research has examined different correlates and predictors for belief in and use of CAM. The findings are varying. In most studies, the predictors were investigated separately (e.g. [2, 4, 8]). Researchers typically focused on a small selection of factors. Thus, there is a particularity—studies so far have investigated relatively small models with only a selected set of predictors in relation to CAM beliefs and beliefs in homeopathy. So far, there is no model for all predictors. This is a gap in the literature. Hence, it is unknown which predictors are strongest—the relative predictive power of each individual predictor remains unknown if it is not assessed in comparison to other potential predictors. Thus, this study is, to the best of our knowledge, a first attempt to investigate most of the variables together along with further variables. The goal of the current study is to test the predictors’ robustness when examined together. We focus on belief since use is not necessarily linked to belief in effectiveness [7].

We aim to obtain insights into key determinants of belief in CAM as well as belief in homeopathy as an explicit and popular form of CAM. Specifically, the present study aims to assess the degree to which belief in CAM and homeopathy is associated with cognitive and personality factors. Our focus is on rather stable individual factors. Certainly, there are also different environments which influence the process of belief and judgement formation resulting in different decisions regarding health products and treatments. However, in this attempt, we aim to find out the individual factors that are more general enduring determinants. These individual differences go beyond specific situations.

The effects are tested in an exploratory manner due to two considerations. First, it is a new approach to analyze most of the predictors in a global analysis. Even if we have some information on the predictors from the reported literature, in these studies, smaller models were used that examined the predictors separately as already mentioned. Therefore, no specific expectations on how the predictive power will change and how strong the predictors will remain in competition with other variables can be derived on this basis. Secondly, we did not preregister hypotheses and the analysis plan. Thus, we follow current recommendations to consider the analyses exploratory (cf. [52–54]).

## Materials and methods

### Sample

Participants were recruited from a large participant pool (*N* = 1,632 participants) established by the University of Hagen, Germany. The personality measures (HEXACO), demographic variables, and level of education were already assessed in a basic survey when registering for the participant pool. 599 individuals participated in the study. Based on a post-hoc power analysis (G-Power [55]) we were able to detect small effects (Cohen’s f² = .02) with 93% statistical power (linear multiple regression, single coefficient, alpha = .05, two-sided test) with this sample size. The sample included 362 women (60%) and 237 (40%) men. Age ranged between 18 und 81 years (*M* = 33.63 years, *SD* = 11.38). Forty-two percent of participants reported holding an academic degree. It was the same sample as in Betsch et al. [48], since the goal of the study was to examine predictors for multiple unfounded beliefs, such as paranormal beliefs, CAM beliefs, and beliefs in conspiracy theories. Predictors for paranormal beliefs were presented in Betsch et al. [48]. Predictors for the other criteria have not yet been analyzed.

### Materials

Causality understanding was assessed with a fictitious scenario called ‘Tom in South America’ (Betsch et al., [Unpublished]). The scenario described an incidence of a seemingly causal relationship. Participants were asked whether the coincidence sufficed as proof of a causal relationship. Three items were based on scientific criteria for verifying a causal relationship, the other three items ignored these criteria and contained information that did not allow causal conclusions. The sum of the correct answers and the correctly rejected answers formed the indicator for the individual causality understanding. The 3-item Cognitive Reflection Test (CRT-3) was used to examine the cognitive style. The test indicated the tendency to override an initial intuitive response by applying analytic thinking skills [40]. It is the most popular instrument based on modern dual process theories of cognition. Epistemological prudence is a concept that describes the testing logic in science and is characterized by critical rationality in thinking [56]. Critical rationalism is one of the epistemological theories by which new knowledge can be gained. It involves forming hypotheses that must be tested for validity. They can be disproved and are thus falsified. An understanding of this testing logic and the realization that the process of gaining knowledge is never complete and scientific results are never final [56] characterizes Epistemological Prudence. It was measured with the Epistemological Prudence Scale (Betsch [Unpublished]). The tendency to see patterns in random events (illusory pattern perception) was examined with random coin tosses as in van Prooijen et al. [43]. Ontological confusions were assessed with the Core Knowledge Confusions Scale [51, 57], which has already been used in previous studies. As in Browne et al. [6], an item combining knowledge and spiritual/religious belief was included in addition to religious belief. The item “The most important knowledge results from religious/spiritual experiences” (7-point Likert scale) should capture an aspect of religiosity/spirituality that is contradictory to an evidence-based world-view, the so-called spiritual epistemology. The personality dimensions openness, emotionality, extraversion, agreeableness, conscientiousness, and honesty-humility were measured with the 100-Item German version of the HEXACO Personality Inventory-Revised (HEXACO-PI-R) [58]. Each dimension consisted of 16 items. Data on numeracy were collected with the DR-Numeracy Test [59]. The ‘Short scale for the assessment of need for cognitive closure’ ([60]; adaption of [61]) was used to assess need for cognitive closure. The ‘Scale for the assessment of need for cognition’ [62] measured need for cognition. To investigate ambiguity tolerance, the ‘Multiple Stimulus Types Ambiguity Tolerance Scale II (MSTAT-II)’ [63] was used. Life satisfaction was assessed with the ‘Short scale life satisfaction-1’ [64]. The Death Anxiety Scale measured death anxiety with 16 items [65]. The socio-demographic variables, precisely age, gender and education, were assessed with self-report items. The key dependent variables CAM belief and belief in homeopathy– a specific form of CAM belief—measured with single items asking for the strength of belief. The item “Please indicate how much you believe in complementary and alternative medicine (e.g., energetic healing, Bach flowers, healing stones …)” as well as the item “Please indicate how much you believe in homeopathy.” were rated on a 6-point Likert scale.

### Procedure

This study was approved by the University of Erfurt Ethic Board and written informed consent was obtained at the beginning of the study. Participants voluntarily agreed to participate in a study on attitudes and beliefs which they could withdraw at any time. The online questionnaire included 121 items in a fixed order. Answering took about 28 minutes. Participants received a flat fee of 5€ in compensation for their participation.

## Results

Reliability of the scales used ranged between.59 and.92 (Cronbach’s alpha). According to conventions, all scales were sufficiently reliable and could be utilized. The exact data is provided in S1 Table.

Overall, we replicated most of the correlations reported in the literature. These findings indicate that the majority of the variables assessed in our study represented promising candidates for predictors of beliefs in CAM and homeopathy. Results are displayed in Table 1.

**Table 1:** Descriptive data and correlations of criteria and potential predictor variables.

Variable	*M*	*SD*	CAM	Homeopathy
*CAM*	3.10	1.68		
*Homeopathy*	3.32	1.70	**.72**	
*Causality understanding*	5.35	1.10	**-.14**	**-.13**
*Cognitive style*	1.75	1.13	**-.13**	**-.16**
*Epistemological prudence*	4.91	.67	**-.10**	**-.16**
*Illusory pattern perception*	2.42	1.32	**.12**	**.15**
*Ontological confusions*	2.47	.59	**.29**	**.29**
*Spiritual epistemology*	2.04	1.37	**.20**	**.13**
*Openness to experience*	3.48	.60	-.05	**-.09**
*Emotionality*	3.23	.59	**.13**	**.10**
*Extraversion*	3.33	.66	.08	**.09**
*Agreeableness*	3.01	.59	.06	.02
*Conscientiousness*	3.57	.57	-.05	-.03
*Honesty* − *humility*	3.58	.72	.00	-.04
*Numeracy*	12.26	1.95	**-.12**	**-.15**
*Need for cognitive closure*	3.24	.70	.04	.02
*Need for cognition*	5.19	.92	**-.11**	**-.15**
*Ambiguity tolerance*	4.49	.75	-.04	-.003
*Life satisfaction*	7.73	2.35	.03	.07
*Death anxiety*	8.04	2.86	**.17**	**.17**
*Age*	33.63	11.38	.03	.02
*Gender*	-	-	**.25**	**.24**
*Education*	-	-	-.08	-.08

Note. *N* = 599. Bold values indicate p <.05.

To assess the degree to which beliefs in CAM and homeopathy are associated with cognitive and personality factors the data were analyzed with a linear multiple forced entry regression analysis containing all of the predictor variables simultaneously. The regression was conducted for CAM belief and for belief in homeopathy each.

As a first step, we checked the assumptions of a regression—they were all met. The correlations between the predictor variables can be found in S2 Table. No variables correlated too highly and the collinearity diagnostics provide no reason for concern (cf. [66]).

The first model tested the predictors in relation to the criterium ‘CAM belief’ resulting in the regression model:

$$\begin{array}{cc} y_{CAM} & =b_{0}+b_{causality\;understanding}*x_{1}+b_{cognitive\;style}*x_{2}+b_{epistemological\;prudence}*x_{3}\\ & +b_{illusory\;pattern\;perception}*x_{4}+b_{ontological\;confusions}*x_{5}+b_{spiritual\;epistemology}*x_{6}\\ & +b_{openness\;to\;new\;experiences}*x_{7}+b_{emotionality}*x_{8}+b_{extraversion}*x_{9}\\ & +b_{agreeableness}*x_{10}+b_{conscientiousness}*x_{11}+b_{honesty/humility}*x_{12}\\ & +b_{numeracy}*x_{13}+b_{need\;for\;cognitive\;closure}*x_{14}+b_{need\;for\;cognition}*x_{15}\\ & +b_{ambiguity\;tolerance}*x_{16}+b_{life\;satisfaction}*x_{17}+b_{death\;anxiety}*x_{18}\\ & +b_{age}*x_{19}+b_{gender}*x_{20}+b_{education}*x_{21}+\epsilon i \end{array}$$

The second model tested the predictors in relation to the criterium ‘belief in homeopathy’ resulting in the regression model:

$$\begin{array}{cc} y_{homeopathy} & =b_{0}+b_{causality\;understanding}*x_{1}+b_{cognitive\;style}*x_{2}\\ & +b_{epistemological\;prudence}*x_{3}+b_{illusory\;pattern\;perception}*x_{4}\\ & +b_{ontological\;confusions}*x_{5}+b_{spiritual\;epistemology}*x_{6}\\ & +b_{openness\;to\;new\;experiences}*x_{7}+b_{emotionality}*x_{8}+b_{extraversion}*x_{9}\\ & +b_{agreeableness}*x_{10}+b_{conscientiousness}*x_{11}+b_{honesty/humility}*x_{12}\\ & +b_{numeracy}*x_{13}+b_{need\;for\;cognitive\;closure}*x_{14}+b_{need\;for\;cognition}*x_{15}\\ & +b_{ambiguity\;tolerance}*x_{16}+b_{life\;satisfaction}*x_{17}+b_{death\;anxiety}*x_{18}\\ & +b_{age}*x_{19}+b_{gender}*x_{20}+b_{education}*x_{21}+\epsilon i \end{array}$$

Table 2 displays the results for both regression analyses.

**Table 2:** Results of regression analysis with cognitive, personality, and socio-demographic variables as predictors as well as CAM belief and belief in homeopathy as criteria.

	Model CAM				Model Homeopathy			
*Predictors*	*b* (*SE*)	*β*	*t*	*p*	*b*(*SE*)	*β*	*t*	*p*
(*constant*)	.71 (1.5)		.48	.63	2.73 (1.50)		1.82	.07
*Causality understanding*	-.09 (.06)	-.06	-1.47	.14	-.07 (.06)	-.04	-1.09	.28
*Cognitive style*	.02 (.07)	.01	.27	.79	.01 (.07)	.01	.14	.89
*Epistemological prudence*	-.03 (.11)	-.01	-.27	.79	-.17 (.11)	-.07	-1.46	.14
*Illusory pattern perception*	.06 (.05)	.05	1.20	.23	.10 (.05)	.08	2.03	**.04**
*Ontological confusions*	.59 (.12)	.21	5.00	**<.001**	.54 (.12)	.18	4.47	**<.001**
*Spiritual epistemology*	.14 (.05)	.11	2.80	**.005**	.05 (.05)	.04	1.00	.32
*Openness to experience*	-.14 (.12)	-.05	-1.22	.22	-.19 (.12)	-.07	-1.57	.12
*Emotionality*	.12 (.13)	.04	.91	.36	.06 (.13)	.02	.47	.64
*Extraversion*	.18 (.11)	.07	1.66	.10	.16 (.11)	.06	1.39	.16
*Agreeableness*	.31 (.12)	.11	2.55	**.01**	.17 (.12)	.06	1.39	.17
*Conscientiousness*	-.05 (.12)	-.02	-.44	.66	.05 (.13)	.02	.38	.71
*Honesty* − *humility*	-.16 (.10)	-.07	-1.65	.10	-.22 (.10)	-.09	-2.17	**.03**
*Numeracy*	-.01 (.04)	-.01	-.32	.75	-.03 (.04)	-.03	-.73	.47
*Need for cognitive closure*	-.16 (.14)	-.07	-1.11	.27	-.29 (.14)	-.12	-2.04	**.04**
*Need for cognition*	-.07 (.09)	-.04	-.76	.45	-.22 (.09)	-.12	-2.29	**.02**
*Ambiguity tolerance*	-.002 (.12)	-.001	-.02	.99	.12 (.12)	.05	.99	.33
*Life satisfaction*	.01 (.03)	.01	.22	.83	.04 (.03)	.05	1.19	.23
*Death anxiety*	.09 (.03)	.15	3.41	**.001**	.10 (.03)	.17	3.94	**<.001**
*Age*	.01 (.01)	.08	1.76	.08	.02 (.01)	.10	2.23	**.03**
*Gender*	.74 (.15)	.22	5.06	**<.001**	.76 (.15)	.22	5.12	**<.001**
*Education*	-.05 (.06)	-.03	-.82	.42	-.05 (.06)	-.03	-.81	.42

Note. *N* = 599. Bold p-values indicate *p* <.05;

Model CAM: *F* (21, 577) = 6.96; *p* <.01; *R* = .45, *R*^2^ = .202, *SE* = 1.53;

Model Homeopathy: *F* (21, 577) = 7.22; *p* <.01; *R* = .46,*R*^2^ = .208, *SE* = 1.55.

The predictor model explained 20.2% of the variance in CAM belief, indicating a high goodness-of-fit [67]. Ontological confusions and spiritual epistemology correlated positively with the criterion. Also, agreeableness and death anxiety were both significant predictors of CAM belief and as well positively correlated. Gender, as a socio-demographic variable, was also positively correlated with CAM belief.

Approximately 21% of the variance in belief in homeopathy was explained with the predictor model, again indicating a high goodness-of-fit [67]. The predictors ontological confusions and illusory pattern perception correlated positively with the criterion. The socio-demographic variables, gender and age, were both positively correlated with belief in homeopathy. Need for cognitive closure, need for cognition, honesty-humility, and death anxiety were significant predictors of belief in homeopathy. Need for cognitive closure, need for cognition, and honesty-humility were negatively correlated with the criterion, whereas death anxiety was positively correlated.

## Discussion

We conducted this study because a considerable number of adults hold beliefs in CAM and homeopathy. This can seem harmless and without any severe consequences, especially in relation to milder diseases—however, this is not the case. Those beliefs rather reflect “a misunderstanding of how evidence for effective treatments is generated and what actually constitutes evidence of efficacy” [2]. Thus, we assessed whether cognitive and personality factors are able to explain differences in CAM belief and belief in homeopathy. Precisely, we investigated the predictors’ robustness when examined together to obtain insights into key determinants associated with beliefs in CAM and homeopathy. There were 21 predictors included in the analyses. According to our findings, individuals with CAM belief were mainly women who tended to confuse ontological categories, acquired knowledge from religious and/or spiritual experiences, had a higher level of death anxiety, and were more agreeable which means, among other things, being lenient in judging others. Further, our results indicate that believers in homeopathy were primarily women with increasing age who also showed ontological confusions, perceived illusory pattern in unrelated stimuli, had a lower need for cognition but at the same time a lower need for cognitive closure and, among other characteristics, had a strong sense of self-importance. They also felt anxious due to death. As we can see, some variables predicted both belief forms, whereas others were predictors of either one belief. Focusing on the cognitive variables, there were biases predicting the beliefs. Those reflect that people do not think scientifically and in adequate categories. People with such cognitive biases apply the wrong distinctive properties to the superordinate categories, base their knowledge on inappropriate foundations, and see relations that might only be due to coincidence. Moreover, gender and age can explain the beliefs as in previous studies [4, 8, 16, 19, 35]. Interestingly, these demographic variables describe a group that is generally more interested in and concerned with health issues and basically engages more with health-related topics [2, 46]. One could argue that the predictors’ informative value is therefore rather limited. On the other hand, they can be valuable in the sense that it can be further evaluated why some—and only some—of those generally interested in health topics turn to CAM and homeopathy. Death anxiety was a strong predictor for both belief forms. This is reasonable, since CAM methods and homeopathic remedies are used to maintain health or advert diseases and physical suffering that might lead to death.

### World view

In general, the results can be seen in terms of a specific perception of the world. A key characteristic of many CAM treatments is the spiritual orientation to knowledge and decision-making [6]. For medical decisions, individuals who agree that important knowledge results from religious or spiritual experiences might not rely on evidence as a proof of efficiency but rather explain it in terms of personal experiences. In a previous study, it was found that some of the primary reasons for the use of homeopathy were having good experiences in the past [19]. Own experiences have a high value and persuasive power but are meaningless from a scientific point of view as individual cases cannot be generalized to others. Another severe problem in relying on personal experiences for proving effectiveness is that there is no control for confounding variables. Thus, experience easily leads to erroneous conclusions about causality due to uncontrolled confounding. This also relates to the predictor illusory pattern perception.

Category mistakes in which properties of living and lifeless entities are mixed (ontological confusions) were a stable predictor not only for CAM beliefs [46] but also for other unfounded beliefs such as paranormal beliefs [48, 51, 57]. The confusions occur between the core attributes of mental, physical, and biological entities and processes. Mixing up those attributes can easily result in incorrect conclusions about treatments, especially considering their physical and biological processes. Paranormal beliefs and CAM belief do not only share ontological confusions as a predictor, paranormal beliefs are typically the best predictor of CAM belief (e.g. [8, 46]). However, comparing those belief forms, it becomes clear that they share many concepts and approaches to explain reality in an unscientific way. Therefore, both belief forms, paranormal beliefs and CAM beliefs, could also be seen as a result of a world view in which scientific evidence is valued less and, instead, emotional and spiritual explanations are consulted.

### Inattention and ignorance

Our results do not replicate previous findings that showed predictive value of certain cognitive variables such as cognitive style (e.g. [4, 6, 7]). An explanation could be that rather inattention to accuracy than inability to consider empirical evidence fosters the beliefs. People might simply not be aware of the absence of evidence. Another possibility is that people are aware of the absence of evidence but are reluctant to engage with it. Practitioners and patients often claim “whatever works is good” or “the main thing is that it works”. Thus, it is ignorance rather than lack of capacity to appropriately process the evidence.

### Limitations

As with most cross-sectional studies using questionnaires, our results are based on self-reports. Additionally, single items were used for measuring belief strength. Even if multi-item measures often have advantages, single items can be advantageous in terms of practical benefits, e.g., adapting to subjects’ limited attention and time resources. There are several single item measures successfully used to measure diverse concepts [68–71] including attitudes [72]. Also, the variance on those items in our sample shows that participants were able to reflect their beliefs and rank them on the scale provided. Another limitation is that the findings are based on regression analyses, which do not provide insight into causality. Thus, the relationship remains correlational. Even if our sample was broader than in many other psychological studies—it was slightly unbalanced, especially in comparison to the German population. It over-represented educated individuals which may led to an inadequate variation of the cognitive variables if we consider the relationship between cognition and education. However, education and the cognitive variables are only weakly correlated. Thus, it can be assumed that the unbalanced sample did not affect the distribution of cognitive variables to a great extent.

## Conclusion

Our findings show that some of the predictors from previous research replicated whereas others did not. Demographics and certain cognitive variables seem to be key determinants associated with beliefs in CAM and homeopathy. Those individual differences and cognitive biases might result in a different perception of the world. However, variables related to abilities did not predict the beliefs. Thus, they might not be a result of inability but rather of ignorance.

## Supporting information

S1 Table Overview of reliability values for the scales used. (PDF)

S2 Table Overview of correlations between the predictors. (PDF)

S1 Dataset Dataset of the sample. (SAV)
