# Supplementary material for: Medical decision making beyond evidence: Correlates of belief in complementary and alternative medicine (CAM) and homeopathy
Source: PLoS One. 2023 Apr 21;18(4):e0284383. doi: 10.1371/journal.pone.0284383 (PMC10121010; doi:10.1371/journal.pone.0284383)
Supplement: S1 Table — (PDF) [file pone.0284383.s001.pdf]

S1 Table

Overview of reliability values for the scales used

Construct & Scale	Cronbach’s α
Causality Understanding: *Tom in South America*	.63
Cognitive Style (CRT3)	.67
Epistemological Prudence: *Epistemological Prudence Scale*	.59
Illusory Pattern Perception: *Pattern in Coin Tosses*	.92
Ontological Confusions: *Core Knowledge Confusions Scale*	.76
HEXACO	
Openness to New Experiences	.67
Emotionality	.63
Extraversion	.78
Agreeableness	.74
Conscientiousness	.71
Honesty-Humility	.75
Numeracy	.59
Need for Cognitive Closure: *NCC-Kurzskala (dt.)*	.83
Need for Cognition: *NFC-K*	.89
Ambiguity Tolerance: *MSTAT-II*	.76
Death Anxiety: *Death Anxiety Questionnaire*	.66

Note. N = 599. The measures for spiritual epistemology, life satisfaction, age, gender, education, and the dependent variables CAM belief and belief in homeopathy consist of only one item.
