# Supplementary material for: Medical decision making beyond evidence: Correlates of belief in complementary and alternative medicine (CAM) and homeopathy
Source: PLoS One. 2023 Apr 21;18(4):e0284383. doi: 10.1371/journal.pone.0284383 (PMC10121010; doi:10.1371/journal.pone.0284383)
Supplement: S2 Table — (PDF) [file pone.0284383.s002.pdf]

S2 Table

Overview of Correlations Between the Predictors.

	Variable	1	2	3	4	5	6	7	8	9	10	11	12	13	14	15	16	17	18	19	20
1.	Spiritual Epist.	—																			
2.	Ontolog. Confusions	**.16**	—																		
3.	Cognitive Style	-.03	**-.22**	—																	
4.	Numeracy	**-.09**	**-.12**	**.41**	—																
5.	Illus. Pat. Perception	**.09**	**.21**	**-.16**	**-.13**	—															
6.	Need for Cognition	-.05	**-.17**	**.20**	**.16**	**-.17**	—														
7.	Need for Cogn. Clos.	.05	**.10**	**-.14**	**-.14**	**.14**	**-.54**	—													
8.	Ambiguity Tolerance	-.05	-.03	**.10**	**.11**	**-.09**	**.48**	**-.69**	—												
9.	Epist. Prudence	**-.11**	**-.28**	**.22**	**.18**	**-.22**	**.35**	**-.28**	**.14**	—											
10.	Causality Underst.	**-.10**	**-.17**	**.14**	**.10**	-.01	**.17**	-.06	.03	**.15**	—										
11.	Honesty-Humility	**.10**	-.02	-.001	.05	-.01	**.15**	**-.17**	.06	**.17**	.04	—									
12.	Emotionality	.06	-.01	**-.15**	-.06	.05	**-.10**	**.26**	**-.24**	.08	-.03	.06	—								
13.	Extraversion	.03	**.10**	.001	-.06	.03	**.13**	**-.27**	**.27**	-.04	-.04	.06	**-.19**	—							
14.	Agreeableness	**.10**	.02	.07	.03	-.01	.07	**-.24**	**.20**	.06	.03	**.27**	**-.18**	**.23**	—						
15.	Conscientiousness	**-.09**	**-.11**	-.05	-.01	-.04	**.23**	-.01	.01	.05	.05	**.20**	.001	**.20**	.01	—					
16.	Openness	.08	**-.11**	**.12**	**.12**	**-.09**	**.31**	**-.26**	**.16**	**.28**	-.05	**.23**	-.02	**.20**	**.08**	**.11**	—				
17.	Death Anxiety	**.13**	.02	**-.11**	**-.10**	.06	**-.14**	**.31**	**-.27**	-.05	-.05	-.05	**.43**	**-.19**	**-.21**	.01	-.03	—			
18.	Life satisfaction	.04	.07	.04	-.001	-.02	**.15**	**-.21**	**.19**	-.05	.001	.06	**-.16**	**.32**	**.13**	**.11**	.002	**-.19**	—		
19.	Age	.07	.04	-.02	.03	**-.10**	**.14**	**-.12**	.004	**.17**	**-.13**	**.18**	**-.14**	.01	-.02	-.02	**.21**	-.07	.04	—	
20.	Gender	**.08**	**.08**	**-.20**	**-.16**	.02	-.07	.08	**-.11**	**.09**	-.04	**.17**	**.38**	-.04	**-.12**	**.10**	.02	**.16**	.02	**-.09**	—
21.	Education[a]	.03	-.06	**.16**	**.13**	**-.11**	**.14**	**-.11**	.04	**.12**	.07	.04	-.03	.01	-.05	.02	.07	-.001	**.12**	**.37**	.01

[a] The data are Spearman's Rho.
